# Validity and Reliability of the Turkish Version of the Sensory‐Motor Dysfunction Questionnaire

**DOI:** 10.1002/brb3.70949

**Published:** 2025-10-20

**Authors:** Mesut Arslan, Yasemin Karaaslan, Zehra Korkut, Seyda Toprak Celenay

**Affiliations:** ^1^ Faculty of Health Sciences, Department of Physiotherapy and Rehabilitation Bitlis Eren University Bitlis Turkey; ^2^ Faculty of Health Sciences, Department of Physiotherapy and Rehabilitation Hatay Mustafa Kemal University Antakya Hatay Turkey; ^3^ Faculty of Health Sciences, Department of Physiotherapy and Rehabilitation Selcuk University Konya Turkey; ^4^ Faculty of Health Sciences, Department of Physiotherapy and Rehabilitation Ankara Yildirim Beyazit University Ankara Turkey

**Keywords:** sensory‐motor dysfunction questionnaire | validity | reliability

## Abstract

**Objective:**

This study aimed to investigate the validity and reliability of the Turkish version of the sensory‐motor dysfunction questionnaire (SMD‐Q‐TR) in individuals with spine pain.

**Methods:**

This methodological study included 133 individuals. The 12‐question SMD‐Q developed to assess sensorimotor dysfunction was translated in accordance with international standards. Pain intensity (neck, low back, back) with the visual analog scale (VAS) and musculoskeletal disorders with the Cornell Musculoskeletal System Discomfort Survey (Cornell) were assessed. For reliability analysis, test–retest reliability with intraclass correlation coefficient (ICC) and internal consistency with Cronbach's α value were assessed. For validity analysis, construct and criterion validity analyses were determined with confirmatory factor analysis (CFA) and correlation tests between the VAS and the Cornell scores, respectively.

**Results:**

The Cronbach's α value of the SMD‐Q‐TR was found to be 0.85. The ICC was also found to be 0.749. According to CFA, it was determined that the model‐data fit was high and therefore it had structural validity. Moreover, weak positive correlations were found between the total score of the SMD‐Q‐TR and the Cornell‐neck/back/spine scores (rho = 0.281; *p* = 0.001, rho = 0.215; *p* = 0.013, rho = 0.368; *p *< 0.001, respectively), whereas moderate and positive correlations were found between the Cornell‐low back/whole body scores (rho = 0.422; *p* < 0.001, rho = 0.408; *p* < 0.001, respectively). In addition, weak, positive correlations were found between the SMD‐Q‐TR total score and the VAS‐neck/low back/back scores (rho = 0.202; *p* = 0.020, rho = 0.317; *p* < 0.001, rho = 0.209; *p* = 0.016, respectively).

**Conclusion:**

The SMD‐Q‐TR was found to be valid and reliable in individuals with spine pain. The use of the scale in clinics may contribute to the early evaluation of sensorimotor disorders seen in spinal problems and to direct patients to appropriate preventive and therapeutic methods. In future studies, the sensitivity, minimal clinical significance value, and cut‐off score of the SMD‐Q‐TR can be calculated.

## Graphical abstract

This graphical abstract summarizes the purpose, methodology, key findings, and conclusions of the validity and reliability study of the Turkish version of the sensory‐motor dysfunction questionnaire (SMD‐Q‐TR).

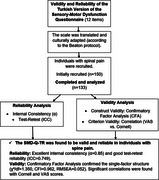

## 1 Introduction

The spine is a complex structure consisting of bone, ligament, muscle, and neural structures, and the holistic interaction between these structures plays a fundamental role in providing axial stabilization. It is also central to the formation of posture and the maintenance of movement capacity (Neumann and Kelly 2010). Incorrect postural habits, prolonged immobility, trauma, and degenerative processes can cause pain in different segments of the spine. These pains, which can be seen from the cervical region to the sacrum, reduce the quality of life and may lead to serious loss of function when they become chronic (Manchikanti et al. 2009).

Spinal pain, including neck and low back pain, is a common musculoskeletal problem and one of the leading causes of disability, contributing to an increased demand for healthcare services worldwide (Vos et al. 2016; Murray et al. 2012). The most common symptom is pain, and this is based on the close anatomical relationship between the spinal cord and vertebrae, discs, ligaments, and synovial joints (Bogduk 1995). The assessment of spinal pain requires a detailed approach due to the complex anatomical and biomechanical structure of the region. Common causes include mechanical stress, degeneration, and inflammation. Acute spinal pain may progress to chronic pain if underlying causes persist or if it fails to resolve despite treatment. In this process, it has been reported that excessive excitability of the central nervous system is effective, and a decrease in gray matter volume has been observed in brain regions related to pain modulation (Kregel et al. 2015). In chronic spinal pain, changes in motor control strategies, cognitive, emotional, and autonomic responses may occur (De Ridder et al. 2022; Jull and Richardson 2000; Lamoth et al. 2006). Spinal muscles may show structural and functional changes in case of injury or pain, which may lead to reorganization in related neural areas when this condition lasts for a long time (Salem et al. 2012; Tinazzi et al. 2000). In particular, impairments in sensory feedback may affect sensorimotor and multimodal integration, resulting in motor control disorders and loss of sensorimotor function (Devecchi et al. 2021; Yu et al. 2021; Desar et al. 2021; Sittikraipong et al. 2020). Sensory‐motor dysfunction is the mismatch between motor intention and sensory feedback. Impaired integration of sensory‐motor information processes may be associated with pain and functional impairment in individuals with chronic musculoskeletal pain (Haavik and Murphy 2012). Therefore, the evaluation of impaired sensory‐motor integration is particularly important in cases of spinal dysfunction. In addition, it should be used in musculoskeletal pain to understand sensory perception and motor control disorders, to direct treatment, and to follow the rehabilitation process.

Ambalavanar et al. (2024, 2025) emphasized the lack of a self‐report measurement tool to assess sensorimotor disorders associated with chronic or recurrent spinal dysfunctions and developed the sensory‐motor dysfunction questionnaire (SMD‐Q) in English to address this need. SMD‐Q is a scale that questions topics such as balance, proprioception, motor performance, and multimodal processing. Limited reliability was observed in the first development study of this scale (Ambalavanar et al. 2024); therefore, the second and third versions were tested with a larger sample, and the third version was determined as the recommended form (Ambalavanar et al. 2025). There is no Turkish version of the SMD‐Q in the literature. The Cornell Musculoskeletal System Discomfort Survey (Cornell), Neck Disability Index (NDI), and Oswestry Disability Index (ODI), which focus on functional limitations rather than sensorimotor integration, are scales that assess sensorimotor disorders (Erdinc et al. 2011; Telci et al. 2009; Yakut et al. 2004). However, the SMD‐Q uniquely assesses balance, proprioception, and multimodal processing, filling a critical gap in the assessment of sensorimotor dysfunction (Ambalavanar et al. 2024).

This study was essential given the absence of validated tools assessing sensorimotor dysfunction in Turkish and the lack of comparable scales internationally. In addition, the assessment of sensorimotor dysfunction is essential for the effective treatment of subclinical cases of spinal dysfunction. Therefore, this study aimed to investigate the validity and reliability of the Turkish version of the SMD‐Q (SMD‐Q‐TR) in individuals with spine pain.

## 2 Materials and Methods

### 2.1 Design

This study employed a methodological study and was conducted in accordance with the principles of the Declaration of Helsinki (Mbuagbaw et al. 2020).

### 2.2 Sampling

In validity and reliability studies, it is recommended in the literature that the sample size should be 5–10 times the number of items in the scale (MacCallum et al. 1999). On the basis of the 12 items in this scale, a minimum sample size of 120 participants was calculated by adopting the upper limit of this recommendation. To account for potential data loss during the collection process, a larger sample size was targeted. Initially, 150 participants were recruited; however, the study was completed with 133 participants, as 17 individuals declined to complete the scales.

### 2.3 Measures

This study adhered to the standardized proposed by Beaton et al. (2000) for translating and culturally adapting the measurement scales. In the first step, the scale was translated from English into Turkish independently by two individuals—one from a healthcare background and one from a non‐healthcare background. In the second step, the Turkish translation was reviewed and discussed by a team of experts in the field, resulting in the first Turkish version of the scale. In the third step, this first Turkish version was back‐translated into English by two independent translators who were fluent in English and were unaware of the original version. In the fourth step, the back‐translated version was compared with the original English scale, and necessary revisions were made to produce a second Turkish version. In the fifth step, this revised version was tested with a group of 30 participants to assess its clarity and comprehensibility. Finally, in the sixth step, the Turkish version of the scale was finalized on the basis of feedback obtained from the pilot testing. No changes were made during the translation process.

After the physical and demographic information of the participants (age, body mass index, gender, smoking/alcohol status, exercise habits) were recorded, the pain localization (neck pain, low back pain, back pain) of all participants was evaluated.

### 2.4 Sensory‐Motor Dysfunction Questionnaire (SMD‐Q)

The SMD‐Q comprises 12 items designed to assess the frequency of impaired sensorimotor functioning, providing an overall indication of sensorimotor dysfunction (Ambalavanar et al. 2025). Participants were asked to choose the option that most accurately reflected their motor behavior. The response options were (1) never/rarely when performing this action/task (<1 day); (2) some or little of the time when performing this action/task (1–2 days); (3) often or a moderate amount of time when performing this action/task (3–4 days of the week); and (4) most or all the time when performing this action/task (5 or more days of the week). Each response was assigned a numerical value ranging from 0 to 3. The total SMD‐Q score was calculated by summing the numerical values across all items, with a maximum possible score of 36. Higher scores reflect more pronounced sensorimotor dysfunction, whereas lower scores suggest minimal dysfunction.

### 2.5 Pain Assessment

Pain localization was assessed using a body pain diagram, whereas pain intensity was evaluated using the visual analog scale (VAS) (Collins et al. 1997). The VAS is a 10‐cm horizontal line representing a continuum from 0 (no pain) to 10 (unbearable pain). Participants were asked to indicate the pain intensity by marking a point on the line that best reflected their experience. The distance in centimeters from the starting point to the mark was measured and recorded as the pain intensity score.

### 2.6 Cornell Musculoskeletal System Discomfort Survey (Cornell)

The Cornell was used to assess the participants’ musculoskeletal system problems (Erdinc et al. 2011). The scale questions the various body regions. It includes questions about how often and how severe pain has been experienced in the body regions in the last week and whether the discomfort prevents work. Individuals are asked to select appropriate options according to the musculoskeletal discomfort experienced in the last week. The person's risk score is calculated with the answers obtained. Pain frequency in the last week is rated as never = 0, once or twice a week = 1.5, three to four times a week = 3.5, once every day = 5, and many times every day = 10, and pain severity is rated as mild = 1, moderate = 2, and severe = 3, and the interference score for work is rated as not hindered = 1, slightly hindered = 2, and very hindered = 3. According to the scoring, each region is scored between 0 and 90. The sections with the highest percentage scores compared to the total score are used to determine the body parts with the most significant problems. The Cornell score was used for the neck, low back, back, spine, and whole body.

### 2.7 Procedures

#### 2.7.1 Data Collection

Participants were reached through the university's online announcement, social media, and e‐mail systems. Verbal and written consents were obtained from individuals who participated in the study. Inclusion criteria were being a native Turkish speaker, being able to read and write in Turkish, having constant or recurring pain, stiffness, or discomfort in at least one of the neck, low back, or back regions for at least 3 months, not having received any treatment in the last month, and being willing to participate in the study. Exclusion criteria were having a neurological disorder, having undergone spinal surgery, and having another painful condition (Ambalavanar et al. 2024, 2025, bib17).

#### 2.7.2 Data Analysis

The conformity of the continuous variables in the study to normal distribution was evaluated graphically and by Shapiro–Wilks test. Mean/Standard deviation (*X* ± SD), number (*n*), and percentage (%) values were used for descriptive data.

For reliability analysis, intraclass correlation coefficient (ICC) results were used for test–retest reliability. Test–retest scores were compared by Wilcoxon signed rank test. In addition, internal consistency was evaluated with Cronbach's α (Cronbach 1951).

Construct and criterion validity analyses were conducted for validity analysis. In this context, confirmatory factor analysis (CFA) was applied to the SMD‐Q‐TR. According to the results of CFA, *χ* ^2^/df (chi‐square statistic/degree of freedom), CFI (comparative fit index), GFI (goodness of fit index), TLI (Tucker–Lewis index), IFI (incremental fit index), and RMSEA (root mean square error of approximation) fit index values were given. In addition, Spearman's rho (*ρ*) coefficient was used in the correlation analysis between SMD‐Q‐TR total score and Cornell pain questionnaire scores (neck, low back, back, spine, and whole body) and VAS scores (neck, low back, back). Correlation coefficient values were determined as 0.0–0.19 very weak, 0.20–0.39 weak, 0.40–0.59 moderate, 0.60–0.79 high, and 0.80–1.00 very high (Prion and Haerling 2014).

IBM SPSS Statistics 21.0 (IBM Corp. Released 2012. IBM SPSS Statistics for Windows, Version 21.0. Armonk, NY: IBM Corp.), AMOS 21.0 programs were used. Statistical significance level was accepted as *p* < 0.05.

## 3 Results

The data of 133 individuals were analyzed in the study. The age of the individuals was 21.23 ± 1.46 years, body mass index was 22.08 ± 3.71 kg/m^2,^ and 106 (79.7%) of them were female. It was determined that 117 (88%) of the individuals had neck pain, 115 (86.5%) had low back pain, and 111 (83.5%) had back pain (Table 1).

**TABLE 1:** Descriptive data of the individuals.

Descriptive data		Mean ± SD (*n* = 133)
Age (years)		21.23 ± 1.46
Body mass index (kg/m^2^)		22.08 ± 3.71
		***n* (%) (*n* = 133**)
Gender	Male	27 (20.3)
	Female	106 (79.7)
Smoking	Yes	38 (28.6)
	No	95 (71.4)
Alcohol use	Yes	7 (5.3)
	No	126 (94.7)
Exercise habits	Yes	33 (24.8)
	No	100 (75.2)
Neck pain	Yes	117 (88)
	No	16 (12)
Low back pain	Yes	115 (86.5)
	No	18 (13.5)
Back pain	Yes	111 (83.5)
	No	22 (16.5)

Abbreviations: kg, kilogram; m, meter; SD, standard deviation.

### 3.1 Reliability Analysis

The corrected item‐total correlation of the scale was calculated, and the item‐total correlation explains the relationship between the scores obtained from the items in the measurement tool and the total score. A high and positive item‐total correlation indicates that the items in the measurement tool exemplify similar behaviors and the internal consistency of the scale is high (Büyüköztürk 2018). It is also stated that item‐total correlations of 0.30 and above are sufficient for the items in the measurement tool and that items with these values are good items (Büyüköztürk 2018; Tavşancıl 2010). The results analyzed in the item‐total correlations for all items were greater than 0.30. When the item‐total correlations are 0.30 and above, it shows that the items measure similar constructs and have internal consistency. Item correlations ranged between 0.376 and 0.708. When the reliability analysis of the scale was examined, Cronbach's α value was found to be 0.850 (Table 2).

**TABLE 2:** Item correlations.

Items	Mean ± SD	Total score	Cronbach's α reliability coefficient with item removed
1	0.53 ± 0.71	0.554	0.833
2	0.63 ± 0.70	0.708	0.827
3	0.47 ± 0.57	0.574	0.844
4	1.01 ± 0.78	0.589	0.846
5	0.32 ± 0.54	0.554	0.837
6	0.52 ± 0.63	0.613	0.839
7	0.26 ± 0.53	0.376	0.851
8	0.41 ± 0.56	0.637	0.833
9	0.38 ± 0.68	0.496	0.841
10	0.31 ± 0.55	0.582	0.838
11	1.08 ± 0.84	0.672	0.838
12	0.38 ± 0.59	0.673	0.831

Abbreviation: SD, standard deviation.

Reliability was assessed through test–retest and ICC. ICC < 0.5 indicates poor reliability, values between 0.5 and 0.9 indicate moderate to good reliability, and values greater than 0.90 indicate excellent reliability (Koo and Li 2016). The ICC value for the SMD‐Q‐TR was 0.749 (*p* < 0.001). In addition, no statistically significant difference was found between the mean scores of the SMD‐Q‐TR total scores at the first and second measurements (*p* > 0.05) (Table 3).

**TABLE 3:** Reliability results for the SMD‐Q‐TR.

	Test Mean ± SD	Re–test Mean ± SD	Intraclass correlation coefficient (ICC)	*p*
SMD‐Q‐TR total score	6.29 ± 4.79	5.64 ± 3.61*	0.749	<0.001

Abbreviations: ICC, intraclass correlation coefficient; SD, standard deviation; SMD‐Q‐TR, the Turkish version of the sensory‐motor dysfunction questionnaire.

*p = 0.432.

### 3.2 Validity Analysis

According to the CFA results, the fit between the model and the data is high. Chi‐square is statistically significant since *p* < 0.001. When this value is divided by the degrees of freedom in order to correct the dependence of the chi‐square value on the degrees of freedom, the value obtained is less than 3 (1.350), indicating model‐data fit. Again, CFI, GFI, TLI, and IFI values of 0.962, 0.928, 0.950, and 0.963, which are indicators of model‐data fit, are indicators of model and data fit. In addition, although the RMSEA value is not greater than 0.05 (0.052), the fact that the RMSEA value covers the value of 0.052 (0.001–0.081) of the 90% probability confidence interval is an indication that the model‐data fit is high (Table 4).

**TABLE 4:** Confirmatory factor analysis fit indexes for the SMD‐Q‐TR.

$\chi^{2}$	SD	$\chi^{2}$/SD	*p*	CFI	GFI	TLI	IFI	RMSEA	90% confidence interval RMSEA
67.511	50	1.350	<0.001	0.962	0.928	0.950	0.963	0.052	0.001–0.081

Abbreviations: CFI, comparative fit index; GFI, goodness of fit index; IFI, incremental fit index; RMSEA, root mean square error of approximation; SMD‐Q‐TR, the Turkish version of the sensory‐motor dysfunction questionnaire; SD, standard deviation; TLI, Tucker–Lewis index; *χ* ^2^/df, chi‐square statistic/degree of freedom.

When all the values related to model‐data fit are taken into consideration, it can be said that the model fits the data satisfactorily; therefore, the scale has construct validity, and the items that make up the Turkish scale evaluate the single‐factor SMD‐Q. When the results of the CFA for the SMD‐Q‐TR are examined, it is seen that the values of the *χ* ^2^/df index, GFI, TLI, IFI perfect fit, and CFI and RMSEA indices have acceptable fit values. When the CFA results for the SMD‐Q‐TR are examined, it is seen that the values of *χ* ^2^/df index, GFI, TLI, IFI perfect fit, and CFI and RMSEA indices have acceptable fit values (Meydan and Şeşen 2011) (Table 5).

**TABLE 5:** Confirmatory factor analysis results for the SMD‐Q‐TR.

Measures of fit	Perfect fit	Acceptable fit	Calculated value	Fit status
$\chi^{2}$/SD	≤3	≤4–5	1.350	Perfect fit
CFI	0.97 ≤ CFI ≤ 1	0.95 ≤ CFI ≤ 0.97	0.962	Acceptable fit
GFI	0.90 ≤ TLI ≤ 1	0.85 ≤ GFI ≤ 0.90	0.928	Perfect fit
TLI	0.95 ≤ NFI ≤ 1	0.90 ≤ NFI ≤ 0.95	0.950	Perfect fit
IFI	0.95 ≤ NFI ≤ 1	0.90 ≤ NFI ≤ 0.95	0.963	Perfect fit
RMSEA	0 < RMSEA < 0.05	0.05 < RMSEA < 0.08	0.052	Acceptable fit

Abbreviations: CFI, comparative fit index; GFI, goodness of fit index; IFI, incremental fit index; RMSEA, root mean square error of approximation; SMD‐Q‐TR, the Turkish version of the sensory‐motor dysfunction questionnaire; TLI, Tucker–Lewis index; *χ* ^2^/df, chi‐square statistic/degree of freedom.

**FIGURE 1:**
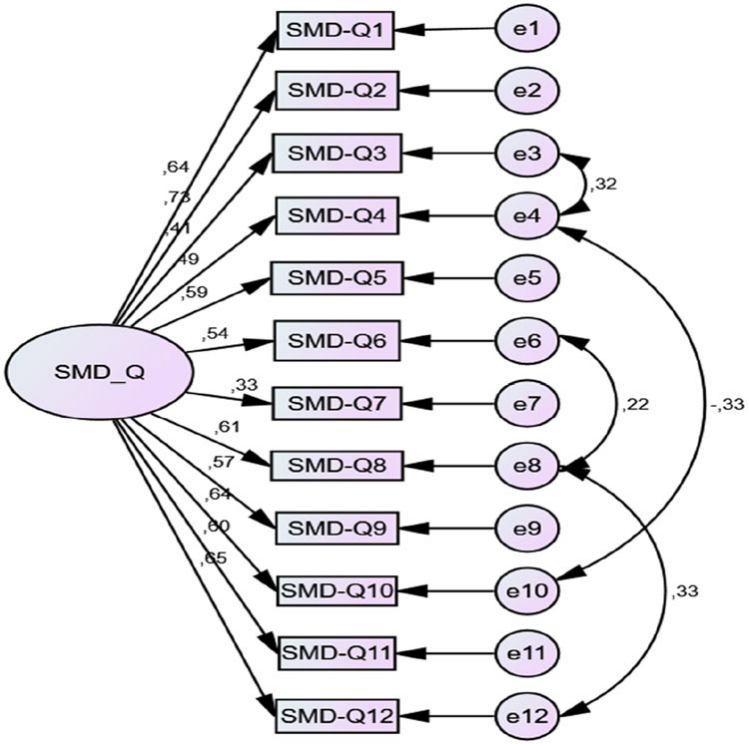
Confirmatory factor analysis diagram for the SMD‐Q‐TR. SMD‐Q, sensory‐motor dysfunction questionnaire.

A weak positive (Fig. 1) and statistically significant relationship was found between SMD‐Q‐TR total score and Cornell‐neck, Cornell‐back, and Cornell‐spine scores (rho = 0.281; *p* = 0.001, rho = 0.215; *p* = 0.013, rho = 0.368; *p* < 0.001). A moderate, positive, and statistically significant relationship was found between SMD‐Q‐TR total score and Cornell‐low back and Cornell‐whole body scores (rho = 0.422; *p* < 0.001, rho = 0.408; *p* < 0.001). A weak, positive, and statistically significant correlation was found between SMD‐Q‐TR total score and VAS‐neck, VAS‐low back, and VAS‐back scores (rho = 0.202; *p* = 0.020, rho = 0.317; *p* < 0.001, rho = 0.209; *p* = 0.016) (Table 6).

**TABLE 6:** The relationship between SMD‐Q‐TR total score and Cornell and VAS scores.

	SMD‐Q‐TR total score	
	rho	*p*
Cornell‐neck	0.281	0.001
Cornell‐low back	0.422	<0.001
Cornell‐back	0.215	0.013
Cornell‐spine	0.368	<0.001
Cornell‐whole body	0.408	<0.001
VAS‐neck	0.202	0.020
VAS‐low back	0.317	<0.001
VAS‐back	0.209	0.016

Abbreviations: Cornell, The Cornell Musculoskeletal System Discomfort Survey; rho, Spearman correlation coefficient; SMD‐Q‐TR, the Turkish version of the sensory‐motor dysfunction questionnaire; VAS, visual analog scale.

## 4 Discussion

This study aimed to investigate the validity and reliability of the Turkish version of the SMD‐Q‐TR in individuals with spine pain. The SMD‐Q‐TR demonstrated excellent internal consistency and good test–retest reliability. CFA confirmed strong structural validity. Significant correlations were observed with Cornell scores, showing moderate associations for low back and whole body and weaker associations for neck, back, and spine. Weak but significant correlations were also found with VAS scores across all spinal regions. This study found that the scale was valid and reliable of the SMD‐Q‐TR in individuals with spine pain.

The SMD‐Q was developed in English by Ambalavanar et al. (2024, 2025). The reliability of the scale was first demonstrated in a pilot study in individuals with spinal pain (Ambalavanar et al. 2024). Then, the second and third repeat reliability of the scale was determined in individuals with subclinical neck pain (Ambalavanar et al. 2025). However, these studies did not include the validity of the scale. In addition, according to the information of the authors, it was seen that the original scale was not adapted to another language, and validity and reliability analyses were not conducted.

It is important for the scale to be valid and reliable in order to standardize it and produce accurate data. Reliability is a necessary prerequisite for validity; a scale cannot be valid if it is not reliable. However, high reliability does not automatically guarantee validity. Therefore, it is crucial to examine both the reliability and the validity of a scale to ensure the accuracy and trustworthiness of the data it produces (Ercan and Kan 2004). In this study, Cronbach's α value for internal consistency in the reliability analysis of the SMD‐Q‐TR was found to be 0.85. In addition, the test–retest reliability of the scale was analyzed, and the ICC was found to be 0.749. This score indicates that the scale has moderate to good reliability. In a pilot study conducted in individuals with spinal pain in the original study, Cronbach's α value was found to be 0.90 (Ambalavanar et al. 2024). In the subsequent study conducted in individuals with subclinical neck pain, Cronbach's α values were 0.74 and 0.89 during the second and third repeat reliability, respectively (Ambalavanar et al. 2025). However, for test–retest reliability, no value was determined according to the total score in the original pilot version of the study (Ambalavanar et al. 2024). Cronbach's α < 0.5 is considered unacceptable, 0.5 ≤ Cronbach's α < 0.6 is considered poor, 0.6 ≤ Cronbach's α < 0.7 is considered doubtful, 0.7 ≤ Cronbach's α < 0.8 is considered acceptable, 0.8 ≤ Cronbach's α < 0.9 is considered good, and Cronbach's α ≥ 0.9 is considered excellent (George and Mallery 2004). An ICC < 0.5 is indicative of poor reliability, those between 0.5 and 0.9 indicate moderate‐to‐good reliability, and values >0.90 indicate excellent reliability (Koo and Li 2016). As a result, it was determined that the SMD‐Q‐TR was reliable for this population.

The other analysis in the evaluation of psychometric properties of a scale was validity. In this study, construct and criterion validity analyses were examined. In this study, the construct validity of the SMD‐Q‐TR was determined by CFA. According to CFA, it was determined that the model‐data fit was high, and therefore, it had structural validity. However, no such data were included in the original version of the studies (Ambalavanar et al. 2024, 2025). It is important to conduct construct validity analysis to determine the ability of a scale to measure the concepts it is intended to measure and to determine what the score obtained as a result of the measurement actually means (Büyüköztürk 2002). The criterion validity of the SMD‐Q‐TR was evaluated with the correlation results between the Cornell‐neck/low back/back/spine/whole body and the VAS‐neck/low back/back scores. According to the correlation results, weak positive correlations were found between the total score of the SMD‐Q‐TR and Cornell‐neck/back/spine scores, whereas moderate positive correlations were found between Cornell‐low back/whole body scores. In addition, weak positive correlations were found between the SMD‐Q‐TR and the VAS‐neck/low back/back scores. Criterion validity was not assessed in the original pilot version of the study (Ambalavanar et al. 2024). Later, in their study in individuals with subclinical neck pain, it was found that there was a positive correlation between the total score of the scale and the VAS score (Ambalavanar et al. 2025). The SMD‐Q‐TR was found to be a valid scale to assess sensorimotor impairments in the Turkish population with spine pain.

A key strength of this study is that, to our knowledge, there is no alternative scale for assessing sensorimotor dysfunction in the literature, and the existing scale lacks established validity and reliability in Turkish. The fact that the sample consisted of young adults may be a limitation of the study. It would be important to also conduct this study in older populations with sensorimotor dysfunction.

## 5 Conclusion

With this study, the SMD‐Q‐TR was found to be valid and reliable in individuals with spine pain. The use of the scale in clinics may contribute to the early evaluation of sensorimotor disorders seen in spinal problems and to direct patients to appropriate preventive and therapeutic methods. In future studies, the sensitivity, minimal clinical significance value, and cut‐off score of the SMD‐Q‐TR can be calculated.

## Author Contributions

**Mesut Arslan**: conceptualization, data curation, formal analysis, funding acquisition, investigation, methodology, project administration, resources, writing – original draft, writing – review and editing. **Yasemin Karaaslan**: conceptualization, data curation, formal analysis, funding acquisition, investigation, methodology, project administration, resources, writing – original draft, writing – review and editing. **Zehra Korkut**: conceptualization, data curation, formal analysis, funding acquisition, investigation, methodology, project administration, resources, writing – original draft, writing – review and editing. **Seyda Toprak Celenay**: Conceptualization, formal analysis, funding acquisition, investigation, methodology, project administration, resources, writing – review and editing.

## Ethics Statement

The present study protocol was reviewed and approved by the Non‐Interventional Clinical Research Ethics Committee of Bitlis Eren University (approval date and number: 05.03.2025‐6).

## Consent

Informed consent was submitted by all subjects when they were enrolled.

## Conflicts of Interest

The authors declare no conflicts of interest.

## Peer Review

The peer review history for this article is available at https://publons.com/publon/10.1002/brb3.70949

## Data Availability

Data available on request due to privacy/ethical restrictions. The data that support the findings of this study are available on request from the corresponding author. The data are not publicly available due to privacy or ethical restrictions.
